# Implementation of Artificial Intelligence in Food Science, Food Quality, and Consumer Preference Assessment

**DOI:** 10.3390/foods11091192

**Published:** 2022-04-20

**Authors:** Sigfredo Fuentes

**Affiliations:** Digital Agriculture, Food and Wine Research Group, School of Agriculture and Food, Faculty of Veterinary and Agricultural Sciences. The University of Melbourne, Parkville, VIC 3010, Australia; sfuentes@unimelb.edu.au; Tel.: +61-3-9035-9670

In recent years, new and emerging digital technologies applied to food science have been gaining attention and increased interest from researchers and the food/beverage industries. In particular, those digital technologies that can be used throughout the food value chain are accurate, easy to implement, affordable, and user-friendly. Hence, this Special Issue (SI) is dedicated to novel technology based on sensor technology and machine/deep learning modeling strategies to implement artificial intelligence (AI) into food and beverage production and for consumer assessment. This SI published quality papers from researchers in Australia, New Zealand, the United States, Spain, and Mexico, including food and beverage products such as grapes and wine [1], chocolate [2], honey [3], whiskey [4], avocado pulp [5], and a variety of other food products [6].

The analysis of big data, such as meteorological and vineyard management information using machine learning algorithms, has been used to target the prediction of aroma profiles for the Pinot Noir cultivar in Australia [1]. Wine aroma and chemometric profile prediction using readily available ancillary information could offer the viticulture and winemaking industries the advantage of characterizing wine regions and specific styles of wine production through vertical vintages. The accuracy of the regression models presented in this paper (*R* > 0.94) can be used to improve or maintain wine quality traits and styles for other wine regions using big data analysis. On the other hand, the quality analysis of chocolate has been based on a digital analysis using machine learning of chemical fingerprinting using near-infrared spectroscopy (NIR) [2]. This paper offered a non-destructive digital method to automatically assess physicochemical and sensory data to potentially achieve digital twins to assess chocolate quality traits more consistently, objectively, and affordably to the industry. The regression machine learning models developed also achieved high accuracy (*R* > 0.93). The classification of unfloral kinds of honey into botanical classes using the standard counting of pollen grains may be a daunting task. Research from Spain [3] proposed using a comparative analysis of a machine learning algorithm’s performances to expedite this classification based on physicochemical parameters obtained from honey samples as inputs and honey classes based on botanical origins as targets. Eleven different ML algorithms were tested, with the penalized discriminant analysis (PDA) being the best performing one for overall accuracy. Interestingly, supervised vector machine (SVM) was the best performing algorithm, which may contradict the use of SVM for other applications published elsewhere. Sensory analysis is an area of research that is increasing the inclusion of digital technologies to assess the subconscious responses of consumers, which can offer a better understanding of the liking and appreciation of different cultures. However, one of the main bottlenecks can be found in the lexicon used when describing food and beverage products. A study from the USA used deep learning algorithms for the sensory descriptor of whiskey lexicon related to flavor characterization [4]. For this purpose, an interactive visual tool was implemented to tag samples of a descriptive lexicon from a database of whiskey reviews. The model proposed was able to identify descriptors with 99% accuracy. This research may facilitate lexicons for other food and beverage products that can also target different cultural backgrounds and perceived terminology that can be equivalent to a more familiar one with specific consumers. Furthermore, the importance of sensory drivers for assessing food products such as avocado pulp of different cultivars has been investigated by a research team in Mexico using predictive modeling strategies [5]. Specific descriptive flavors, textural sensory drivers, instrumental stickiness, and color were found within the map modeling strategy implemented that can be useful for selecting avocado fruits to develop particular products with maximum acceptability by consumers. Finally, a research group from Mexico studied the digital assessment of consumers’ physiological responses to sensory analysis of different products, including facial emotional recognition, galvanic skin response, and heart rate [6]. The integration of different sensors and analysis using machine learning algorithms targeted consumer acceptability with the best prediction, compared to the use of individual sensor technologies. The authors proposed using integrative biometric systems to completely predict the sensory responses using physiological responses alone to assess new food products. 
